# Gradient-doped SnO_2_ for perovskite photovoltaics

**DOI:** 10.1016/j.xinn.2026.101544

**Published:** 2026-08-27

**Authors:** Jiahui Qi, Wenjie Yang, Hieng Kiat Jun, Zhong Chen, Guohua Xie, Jianghui Zheng

**Affiliations:** 1 School of Electronic Science and Engineering, Xiamen University, Xiamen 361005, China; 2 Shenzhen Research Institute of Xiamen University, Shenzhen 518057, China; 3 Department of Mechanical and Materials Engineering, Universiti Tunku Abdul Rahman, Sungai Long Campus, Kajang 43000, Malaysia; 4 Institute of Flexible Electronics (IFE, Future Technologies), Future Display Institute of Xiamen, Tan Kah Kee Innovation Laboratory, Xiamen University, Xiamen 361102, China; 5 Centre for Sustainable Mobility Technologies, Universiti Tunku Abdul Rahman, Sungai Long Campus, Kajang 43000, Malaysia; 6 Centre for Advanced and Sustainable Materials Research, Universiti Tunku Abdul Rahman, Sungai Long Campus, Bandar Sungai Long, Kajang 43000, Malaysia

## Main text

Although single-junction perovskite solar cells (PSCs) are rapidly approaching the Shockley-Queisser limit, a persistent efficiency gap remains between the conventional (n-i-p) and inverted (p-i-n) architectures. While inverted devices have achieved a certified power conversion efficiency (PCE) of 28.0%,^1^ the certified PCE of conventional n-i-p PSCs has long stagnated near 26%. This discrepancy no longer originates from the perovskite absorber itself but increasingly from the electron-transport interface. However, tin oxide (SnO_2_) in conventional n-i-p PSCs still suffers from limited control over interfacial electronic properties and defect distribution.^2^ Against this backdrop, SnO_2_ deposited using the chemical-bath-deposited (CBD) method can achieve uniform coverage on textured substrates, and with its low-temperature processing and low cost, it is an ideal choice for realizing high-performance n-i-p devices.

## Bottlenecks in CBD-SnO_2_

Despite its advantages in conformal deposition and industrial compatibility, CBD-SnO_2_ suffers from a fundamental limitation: the electronic structure of the film is difficult to precisely regulate during growth. During the CBD process, the oxidation kinetics of Sn^2+^ precursors strongly influence oxygen-vacancy (*V*_O_) formation and defect distribution. Notably, *V*_O_s in SnO_2_ can act as shallow-level donor defects, increasing electron density and electrical conductivity. However, when their concentration is too high, they introduce defect states that promote nonradiative recombination and accelerate material degradation. Therefore, for CBD-SnO_2_, the challenge lies not in maximizing or eliminating *V*_O_s but in balancing their beneficial and harmful effects. Furthermore, hydroxyl groups, residual ligands, and dangling bonds enriched on the SnO_2_ film surface introduce deep-level defects into the band gap, acting as Shockley-Read-Hall recombination centers that directly deplete photo-generated carriers. Consequently, conventional CBD-SnO_2_ still faces intrinsic bottlenecks in enabling high-performance and stable PSCs.

## Toward graded CBD-SnO_2_

Previous studies have explored various optimization approaches. For example, Lin et al. proposed a pre-nucleation strategy to achieve SnO_2_ deposition driven by single-site heterogeneous nucleation, enabling carbon-based PSCs without a hole transport layer to achieve a record efficiency of 21.01%.^3^ Seo et al. obtained tin oxide films with complete surface coverage and low defect density through an excess ligand strategy, thereby reducing surface reflectance and improving charge extraction, ultimately increasing the certified efficiency of single-junction PSCs to 26.10%.^4^ Although CBD of SnO_2_ is the benchmark method for fabricating state-of-the-art n-i-p PSCs (including large-area devices and modules), it offers limited control over electronic properties. Previous research strategies have primarily focused on optimizing film formation, surface coverage, and defect passivation, viewing *V*_O_s as harmful defects while overlooking the fact that they are the main source of n-type doping in oxidized tin. Consequently, there is an urgent need to elucidate the underlying formation mechanisms and develop advanced strategies to drive the advancement of n-i-p PSCs.

Against this backdrop, Wang et al. found that the performance bottleneck in textured substrates stems from a synergistic effect between band misalignment and electron accumulation at the buried substrate interface.^5^ Further studies indicate that the incomplete oxidation of the Sn^2+^ precursor to Sn^4+^ during the CBD process is the key factor driving the incorporation of *V*_O_ into the tin oxide lattice. This insight motivated the authors to fundamentally revisit the formation mechanism of CBD-SnO_2_ films and the microscopic origin of their electronic heterogeneity. Through combined characterization using cross-sectional transmission electron microscopy (TEM) and time-of-flight secondary ion mass spectrometry (TOF-SIMS), it was revealed that CBD-SnO_2_ is not simply a continuous dense film but rather a quasi-layer-by-layer (LBL) assembly of monodisperse colloidal nanoparticles (NPs) with diameters of 3–5 nm, formed through electrostatic adsorption and LBL stacking. This structural picture provides a mechanistic basis for spatially graded electronic structure design.

## Ligand-controlled gradient doping

A key aspect of this ligand-controlled gradient doping strategy is the regulation of *V*_O_ incorporation during CBD-SnO_2_ growth through ligand modulation. Sulfoacetic acid (SA) promotes the formation of SnO_2_ NPs (n^+^) that are rich in *V*_O_s and have high electron density, whereas phosphonoacetic acid (PA) facilitates more complete oxidation, resulting in lightly doped particles (n). This dynamic evolution of NP doping characteristics lays the foundation for constructing a continuous n^+^/n gradient structure. As shown in Figure 1, since the binding energy of PA to the SnO_2_ surface (3.99 eV) is significantly higher than that of SA (2.67 eV), PA thermodynamically dominates competitive adsorption and preferentially displaces SA. This demonstrates that the direct blending of different ligands is insufficient to spontaneously generate a spatially continuous gradient. Therefore, Wang and co-workers ingeniously substituted PA with its precursor, 2-(dimethoxy phosphoryl) acetic acid (DMPA), utilizing its controllable hydrolysis kinetics to achieve controlled release of PA. During the initial deposition stage, both SA and DMPA are present in the chemical bath. However, because DMPA hydrolyzes slowly, the concentration of released PA remains negligible, allowing SA to dominate surface coordination and promote the formation of highly doped n^+^ NPs. As DMPA gradually hydrolyzes, the PA concentration accumulates, achieving a ligand transition at the competition threshold and ultimately forming a lightly doped n-type layer. This continuous n^+^/n gradient creates a built-in electric field directed toward the cathode, effectively resolving the long-standing trade-off between high contact conductivity and minimized interfacial band offset that cannot be simultaneously achieved in uniformly doped SnO_2_ layers. These results highlight the spatial continuity of the functional gradient as a critical design parameter for suppressing interfacial losses. More importantly, this strategy converts ligand chemistry from a passive surface modification tool into an active regulator of spatially resolved electronic structure.

**Figure 1:**
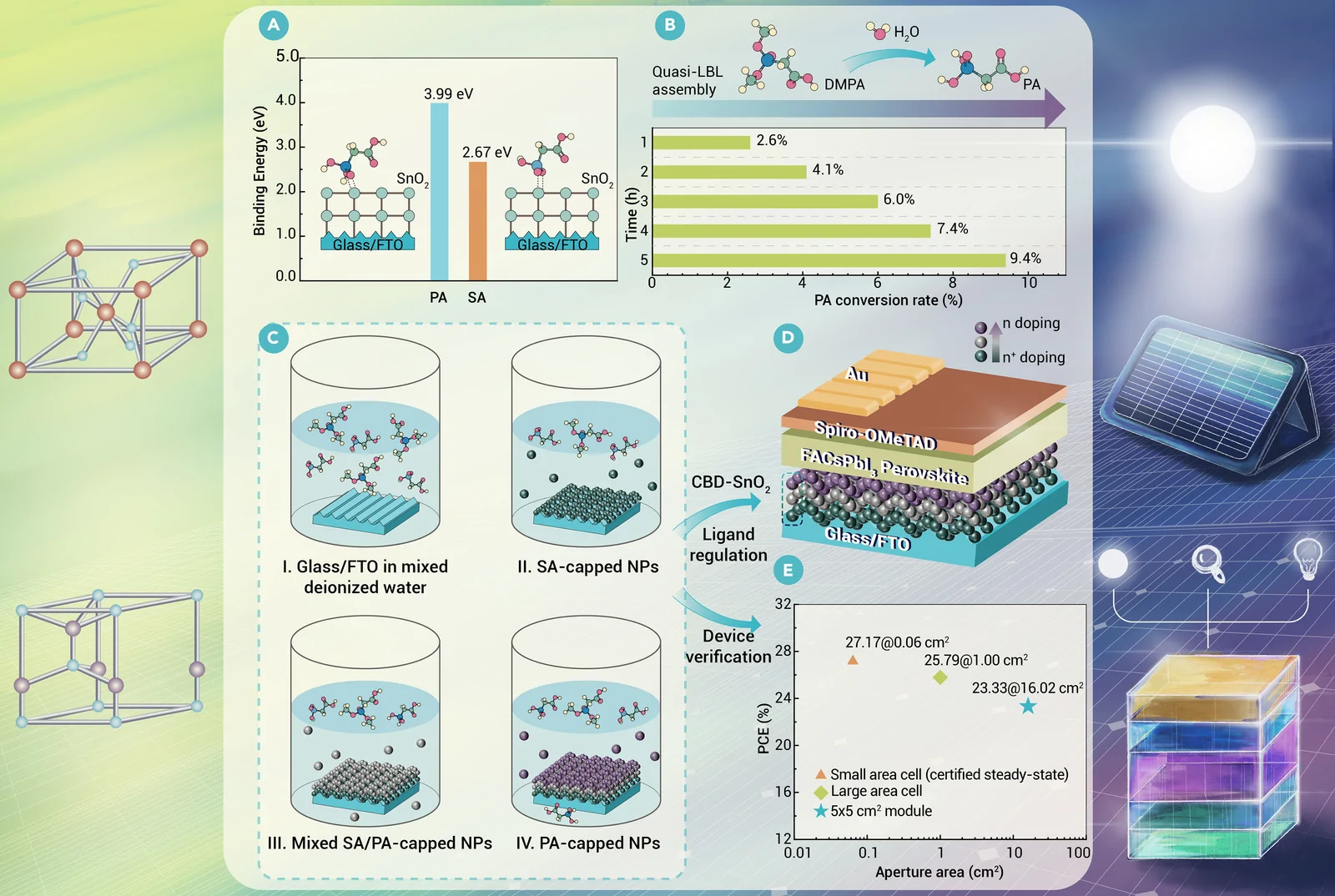
Process and applications of chemical-bath-deposited gradient-doped SnO_2_ for perovskite photovoltaics (A) Calculated binding energies of SA and PA ligands on SnO_2_ surfaces. (B) Conversion of DMPA to PA at different retention times during the CBD process. (C) Schematic illustration of continuously graded-doped SnO_2_ film construction via controlled hydrolysis. (D) Schematic of n-i-p device configuration incorporating a continuously graded-doped SnO_2_ electron transport layer (ETL). (E) Device performance of perovskite solar cells incorporating continuously gradient-doped SnO_2_ ETLs at different aperture areas.

## Device-level validation

Spatially continuous gradient doping not only reduces the conduction-band offset and optimizes band alignment but also accelerates charge extraction and eliminates interfacial carrier accumulation through an intrinsic electric field, transforming these two previously conflicting physical parameters into synergistic design variables. Upon integration into fully functional PSCs, independent certification by the National Photovoltaic Industry Metrology and Testing Center (NPVM, China) confirmed a certified steady-state PCE of 27.17%, establishing a new efficiency record for the single-junction n-i-p architecture. The simultaneous increase in open-circuit voltage (*V*_OC_) and fill factor is consistent with effectively suppressed interfacial nonradiative recombination. The ideality factor, as measured from light-dependent *V*_OC_ measurements, approaches 1, further confirming the suppression effect of gradient doping on interfacial recombination. Furthermore, large-area 1 cm^2^ devices and 5 × 5 cm^2^ modules (aperture area 16.02 cm^2^) achieved PCEs of 25.79% and 23.33%, respectively, demonstrating the scalability of the ligand-competitive binding strategy toward large-area perovskite photovoltaics. Importantly, because the electronic structure gradient is formed intrinsically during the CBD growth process, this strategy combines advanced interfacial control with the inherent scalability and manufacturability of CBD SnO_2_. Standard stability tests (ISOS protocol) also confirmed that the gradient architecture does not compromise the device’s long-term operational reliability.

## Concluding remarks

Fundamentally, in n-i-p-type photovoltaic devices, this study shifts the optimization paradigm for CBD-SnO_2_ from empirical solution bath engineering to programmable electronic structure design. Spatially modulating the electronic structure of SnO_2_ simultaneously meets the requirements for low contact resistance on the cathode side and a low band offset at the buried perovskite/SnO_2_ interface. As perovskite photovoltaic technology approaches its practical efficiency ceiling, interfacial carrier losses are expected to become an increasingly dominant factor governing device performance. This electronic structure modulation is directly integrated into the CBD-SnO_2_ growth process, preserving the inherent scalability and manufacturability of CBD while providing a highly promising path toward high-efficiency, large-area devices and modules. Beyond PSCs, this concept may also be applicable to other solution-processed metal oxide transport layers, such as titanium dioxide (TiO_2_) and zinc oxide (ZnO), as well as more complex device architectures, including perovskite tandem solar cells and light-emitting diodes. More broadly, a spatially programmable transport layer could serve as a versatile strategy for overcoming interface limitations in solution-processed semiconductor devices.

## Funding and acknowledgments

This work is supported by the 10.13039/501100001809National Natural Science Foundation of China (62404185) and the 10.13039/501100021171Guangdong Basic and Applied Basic Research Foundation (2026A1515030023). J.Z. acknowledges support from the Nanqiang Outstanding Young Talents Program of Xiamen University (X2450215).

## Declaration of interests

The authors declare no competing interests.
